# Substance misuse teaching: a patient safety issue

**DOI:** 10.1192/bjo.2021.514

**Published:** 2021-06-18

**Authors:** Lucy Harborow, Mary Thornton, Nicola J. Kalk, Mike Kelleher

**Affiliations:** 1 South London and Maudsley NHS Foundation Trust, Kings College London; 2 South London and Maudsley NHS Foundation Trust, Kings College London, Kings College London; 3 South London and Maudsley NHS Foundation Trust, Kings College London, Institute of Psychiatry, Psychology and Neuroscience, King's College London

## Abstract

**Aims:**

Clinical substance misuse presentations are commonly managed by Psychiatry Core Trainees (CTs) out of hours. However, specialist teaching is not included in the Maudsley Training Program (MTP) induction. We aimed to investigate whether this was of clinical concern and, if so identify interventions to address it.

**Background:**

The association of substance misuse disorder and mental illness is widely recognised. The Adult Psychiatric Morbidity Survey 2014 reported that half of people dependent on drugs other than cannabis were receiving mental health treatment. Substance use substantially impacts clinical risk; 57% of patient suicides in 2017 had a history of substance misuse. It also effects emergency psychiatric services: 55-80% of patients detained under S136 are intoxicated. Therefore, it is imperative for patient safety that CTs can assess and manage these patients appropriately.

The Royal College of Psychiatrists recognises the need for specialist substance misuse knowledge and skills, and lists this as a key ‘Intended Learning Outcome’ for CTs. Unfortunately, the availability of specialist drug and alcohol service placements for CTs has significantly declined. Only one placement is available per MTP rotation. Teaching is therefore relied upon to gain these competencies.

**Method:**

Using a cross-sectional survey we explored CTs confidence in recognising and managing substance misuse presentations, knowledge of where to seek guidance and asked for teaching suggestions. We surveyed two CT1 cohorts in 2017 and 2019.

**Result:**

Fifty-one CTs took the survey. Of these 92% did not feel prepared to manage acute substance intoxication or withdrawal and 96% would like relevant teaching at the start of CT1. Furthermore, 67% did not know where they could seek guidance.

CTs felt confident at recognising and managing alcohol related presentations. However, they were less confident in recognising opioid withdrawal, how to safely prescribe opioid substitution therapy (OST), and the usual doses of OST (65%, 94%, 94% rated ‘neither confident nor not confident’ or below, respectively). CTs were not confident at recognising GBL and cannabinoid withdrawal, principles of harm minimisation, assessing readiness to change, delivering Brief Interventions and teaching patients to use Naloxone.

**Conclusion:**

The results were exceptionally similar between cohorts, demonstrating reliability of our findings and that CTs lack of substance misuse knowledge is a significant clinical concern.

To address this deficit of knowledge, we are writing an introductory lecture with supporting guidance in the induction pack, developing an online video resource, and moving key substance misuse lectures to earlier in the MTP taught programme.

